# Temperament and adolescent suicide attempts: a case-control study with multi-ethnic Asian adolescents

**DOI:** 10.1186/s12888-023-04914-6

**Published:** 2023-06-15

**Authors:** Sean H.Y. Toh, Michelle J.S. Wan, Leoniek M. Kroneman, N. Nyein, John C.M. Wong

**Affiliations:** 1 NUS Mind-Science Centre, Department of Psychological Medicine, Yong Loo Lin School of Medicine, National University of Singapore, Queenstown, Singapore; 2 Department of Psychological Medicine, National University Health Systems, Queenstown, Singapore; 3 Research Department, Intellect Pte Ltd, Tanjong Pagar, Singapore

**Keywords:** Temperament, Suicide attempts, Adolescence, Risk factors, Protective factors, Psychiatric Disorders, Comorbidity, Stressful life events, Parental rejection, Asia

## Abstract

**Background:**

Suicide is the leading cause of death for adolescents in several parts of Asia, including Singapore. This study examines the relationship between temperament and youth suicide attempts in a sample of multi-ethnic Singaporean adolescents.

**Methods:**

A case-control design compared 60 adolescents (*M*_*age*_ = 16.40, *SD*_age_ = 2.00) with a recent suicide attempt (i.e., past 6 months) with 58 adolescents (*M*_*age*_ = 16.00, *SD*_age_ = 1.68) without any history of suicide attempts. Presence of suicide attempts was established using the semi-structured interviewer-administered Columbia Suicide Severity Rating Scale. Participants also completed self-report measures on temperament traits, psychiatric diagnoses, stressful life events, and perceived parental rejection in an interview-based format.

**Results:**

Psychiatric comorbidity, recent stressful life events, perceived parental rejection, and all five “difficult temperament” traits, were significantly overrepresented among adolescent cases relative to healthy controls. Adjusted logistic regression models revealed significant associations between suicide attempt, MDD comorbidity (OR: 10.7, 95% Cl: (2.24–51.39)), “negative mood” trait (OR: 1.12–1.18, 95% Cl: (1.00–1.27)), and the interaction term of “positive mood” and “high adaptability” traits (OR: 0.943 – 0.955, 95% Cl: (0.900 − 0.986)). Specifically, “positive mood” predicted lower likelihood of a suicide attempt when “adaptability” was high (OR: 0.335 – 0.342, 95% Cl: (0.186 − 0.500)) but not low (OR: 0.968 – 0.993, 95% Cl: (0.797 − 1.31)).

**Conclusion:**

Temperament screening may be important to identify adolescents at higher or lower risk of suicide at an early stage. More longitudinal and neurobiological research converging on these temperament findings will be helpful in ascertaining temperament screening as an effective suicide prevention methodology for adolescents.

## Introduction

Adolescent suicide is universally recognized as a public health crisis. In Asia, suicide ranked the first leading cause of death for youths aged 10–19 among several countries (South Korea, [1]; China, [2]; Japan, [3]; Singapore, [4]). Though suicide attempts (SAs) are rare in childhood (i.e., ≤ 13 years old), they increased dramatically through adolescence [5, 6]. During this sensitive period of development, having a difficult temperament [7], a major depressive disorder (MDD) diagnosis [8] or comorbidity [9], experiencing stressful life events [10] or parental rejection [11] posed significant, independent risks to SA. Of which, temperament has garnered much attention in recent years for several reasons. First, a “difficult temperament” was found to elevate an adolescent’s susceptibility to these other SA-related risk factors. Traits remarked to be “difficult” in Western cultures, namely negative affectivity, low adaptability/inflexibility to environmental changes, high withdrawal from novelty, high activity levels, and reduced biological rhythmicity, either independently or interact to incline SA-related risks [12]. For example, the propensity to experience frequent, negative affect is central to the tripartite model of depression [13]. Combining this with an inflexible temperament, they increased the likelihood of alcohol and drug addictions through exacerbated emotional difficulties [14]. Biological irregularity and high activity levels in children can disrupt mutually supportive relationships with their parents, which later translates to difficulties in receiving parental acceptance during adolescence [15]. Highly withdrawn adolescents encountered more stressors in school and at home due to their deficits in social functioning [16]. Beyond the “difficult” profile as a predisposing risk factor, specific temperament traits additionally served as direct predictors or protective factors of adolescent SAs. Anxious, depressive, cyclothymic, and irritable styles of temperament, which were highly related to the temperament trait of “negative affectivity/mood” [17, 18], uniquely predicted SAs across all age groups [19]. The “negative mood” trait retained its predictive power even after controlling for psychiatric disorders (MDD, substance use), childhood sexual abuse, an inflexible temperament, and gender [20]. Currently, these robust findings were not observed among other “difficult” traits [20–22]. Conversely, a growing body of research found a hyperthymic temperament to be uniquely associated with reduced likelihood of SAs even after accounting for multiple protective factors [23–27]. Despite being a multifaceted temperament, researchers suggested that the sustained positive affectivity within this profile was largely responsible in providing effective defences against suicidal impulses [28].

As these findings were mainly derived from Western samples, researchers have challenged their generalizability to Asian populations [29]. Culturally and universally, we believe that three areas about temperament as an indirect and direct factor linking to SAs remained relatively understudied: (a) Between individualistic and collectivistic cultures, there were self-reported differences on what constitutes a “difficult” temperament. For instance, while “highly withdrawn” adolescents in Canada typically faced maternal and peer rejection, their counterparts in China experienced entirely opposite outcomes [30, 31]. Among Chinese adolescents, this trait further predicted other positive adjustments including teacher-assessed competence, leadership, academic achievements, self-efficacy, and lower feelings of loneliness and depression [32]. Possibly, this is because “shyness” was commonly perceived as an expression of social maturity and competence in interdependent societies [33]. In contrast, risks associated with the remaining “difficult” traits seemed to apply universally (see [34] for a review). A careful re-evaluation of Western definitions of a “difficult temperament” and its relation to Asian adolescent SAs may be helpful so as to not misinform early screening practices in Asia. (b) Despite existing evidence on the “negative mood” trait being a robust direct predictor, few studies have comprehensively assessed and controlled for other SA-related risk factors that were also influential during adolescent development (i.e., MDD morbidity and comorbidity, proximal stressful life events, perceived parental rejection, other difficult temperaments). (c) Recent studies investigating the protective role of hyperthymic temperament against adolescent SAs yielded mixed findings, either by failing to observe any significant associations [35] or even observing the inverse relationship [36, 37]. For example, Karam et al. (2015) discovered three risk facets underlying a hyperthymic temperament, including “liking to be the boss”, “getting into heated arguments”, and “the right and privilege to do as I please”. One suggestion to reconcile these findings may be to consider the degree of “adaptability” within this profile. Adolescents with low flexibility/adaptability temperaments were more likely to exhibit self-centeredness, higher impulsivity, and confrontational behaviours [38], resembling the three risk facets. Additionally, adolescents with temperaments of positive affectivity but low adaptability previously attempted suicide [39]. This finding challenged previous suggestions that sustained positive affectivity was the main protective factor underlying a hyperthymic temperament. High mood and adaptability were two traits previously associated with high self-confidence [40], which Karam et al. (2015) also found to be the only protective item against SA in hyperthymic female adults. Given these findings, the interplay between “positive affectivity” and “high flexibility/adaptability” may serve as one possible protective pathway against adolescent SAs.

The present study evaluated the risk and protective temperaments among Asian adolescents with previous suicide attempts, according to the following hypotheses: First, contemporaneously with other risk factors (i.e., MDD, other psychiatric disorders, recent stressful life events, parental rejection), a “difficult temperament” but without “high withdrawal” would be significantly overrepresented among Asian adolescent suicide attempters relative to non-attempters. Second, following earlier findings, we expected the “negative affectivity/mood” temperament trait to remain a robust predictor of SA even after controlling for other risk factors. Third, we hypothesized a significant interaction between “positive affectivity/mood” and “adaptability” traits, such that high levels of positive mood and adaptability would be significantly associated with a reduced likelihood of SAs suicide attempts.

## Methods

### Participants and procedures

Ethics approval was obtained from the Domains-Specific Review Board (DSRB) at a large teaching hospital (i.e., National University Hospital) in Singapore. Recruitment procedures for this study lasted a total of 20 months before the COVID-19 pandemic. The inclusion criteria for cases were adolescents aged 13–19 who were admitted to the National University Hospital’s Emergency Department for a recent SA (i.e., over the last 6 months). The inclusion criteria for controls were same-age adolescents who were admitted to the National University Hospital either (i) for an elective surgery or (ii) for having acute medical conditions without severe morbidity which may not require an elective surgery. Controls who have (i) attempted suicide or self-harm at least once in their lifetime, and/or (ii) suffered from significant physical or clinical morbidity were excluded. This exclusion criteria only applies to control subjects as we intended to approximate them to healthy youths within the community. Control subjects were matched as closely as possible to cases by demographic variables (i.e., age, sex, ethnicity). All adolescents who met the above eligibility criteria as a case or control, along with their accompanying parent(s)/guardian(s), were briefed by their attending doctors on the study’s objectives on the day they were admitted to the ward. These attending doctors then sought the permission of both the adolescent and the parent(s)/guardian(s) to be referred to the study team if the adolescent showed interest to participate in the study. If the parent(s)/guardian(s) of the interested adolescent was not present during the day of admission, the doctors would contact the parent/guardian separately over a phone call to describe the study and seek his/her permission to be referred to the study team. When permission was granted by both the adolescent and the parent(s)/guardian(s), the trained research assistants contacted both parties over phone to schedule an in-person interview at the convenience of all personnels. Over these phone calls, the research assistants also explained that the interview with the adolescent may last for two hours, should informed consent be obtained from both the parent/guardian and the adolescent before the interview commences. All interviews were scheduled within the duration of each adolescent’s ward stay and conducted in their wards. These interviews were conducted over a few days to a week after the initial recruitment. Before the start of the interview, the research assistants refreshed the study’s main purposes and procedures for the adolescent and the parent(s)/guardian(s). After obtaining written informed consent from the parent(s)/guardian(s) and the adolescent himself/herself, the interview proceeds between two trained research assistants and the adolescent for the next two hours during which the adolescent completed an interviewer-administered questionnaire (i.e., Columbia Suicide Severity Scale/C-SSRS; see Sect. 2.2.2) and several self-reports. On average, administration of the C-SSRS took 35 min while completion of the remaining self-reports took 85 min. Fifteen-dollar supermarket vouchers were reimbursed for complete responses. For each adolescent who did not complete the full set of questionnaires due to other reasons, s/he will be reimbursed with a five-dollar supermarket voucher for partial response.

**Fig. 1:**
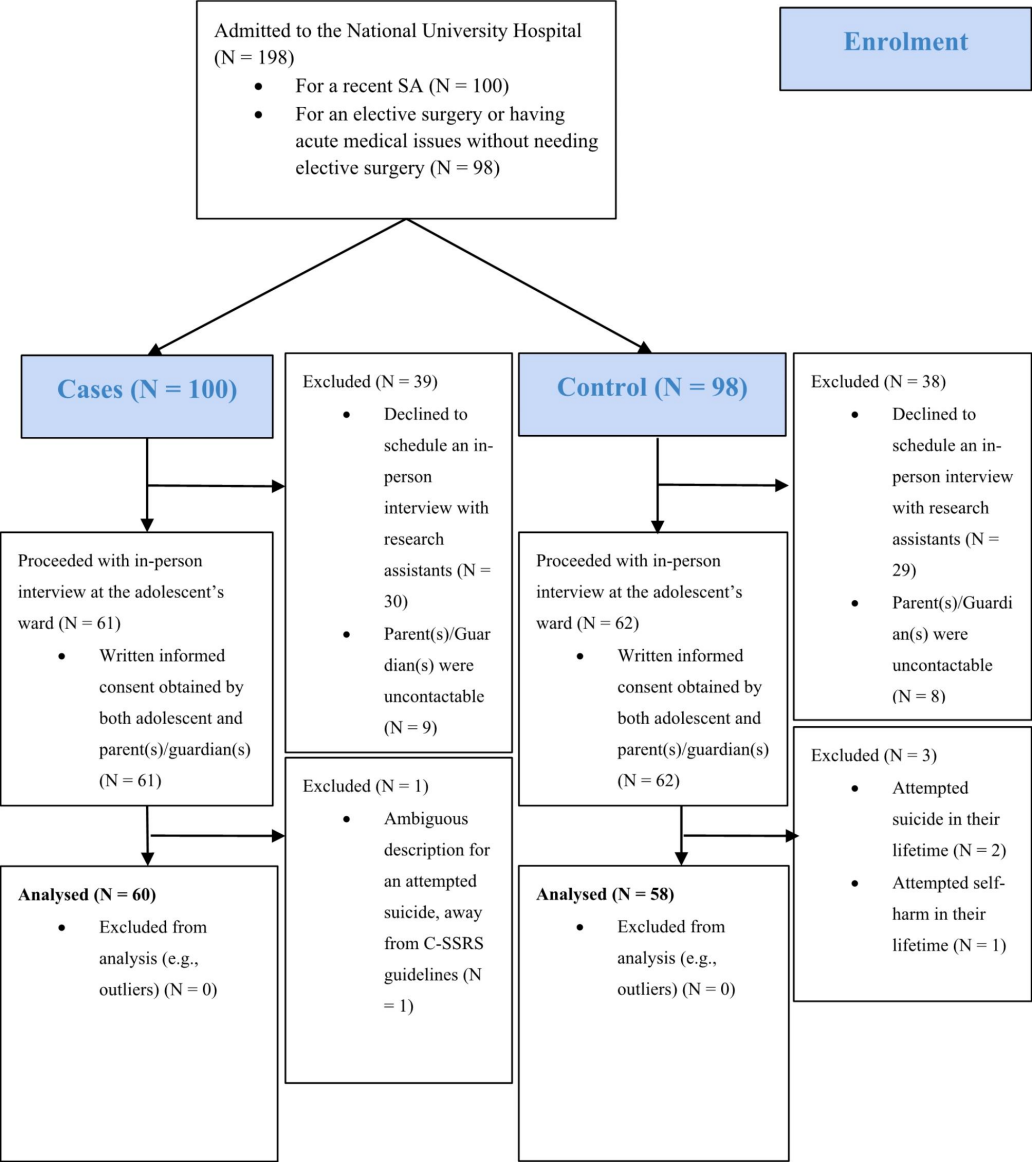
CONSORT Flowchart

Figure 1 illustrates the CONSORT diagram for this case-control study. Over the 20-month recruitment window, a total of 198 eligible adolescents were admitted to the National University Hospital’s Emergency Department and referred by the attending doctors to the study team. Of these 198 adolescents, 59 of them (30 cases, 29 controls) further declined to schedule an in-person interview with the research assistants. Among the remaining 139 adolescents, permission could not be sought from their parent(s)/guardian(s) for 17 of them (9 cases, 8 controls) as they were uncontactable. Informed consent was obtained from the remaining 122 adolescents (61 cases, 61 controls) and his/her parent(s) before the commencement of their interviews. Partial responses were obtained from 1 case subject and 3 control subjects. These 3 control subjects disclosed previous SAs or self-harm during the administration of the C-SSRS, rendering them unfit as a healthy control for this study. The only case subject described his/her SA as knocking an arm against the wall repetitively, which did not fit into the C-SSRS’s recommended description of a typical SA [41]. As the C-SSRS was the first item to be administered in the interview, these adolescents did not continue with the interview. Subsequently, these responses were excluded from the analyses. Together, the response rate achieved was 65.6% for cases and 60.6% for controls respectively. Our final sample comprises 118 adolescents with complete responses (60 cases, 58 controls, M = 16.20, SD = 1.852), with the majority being Chinese (51%) and Females (72%).

All of the research assistants were trained in consent taking procedures and administration of the semi-structured interview and self-reports. Before recruitment commences, the research assistants underwent one full day of training and supervision conducted by author John C.M. Wong who is a certified child and adolescent senior consultant psychiatrist with more than 20 years of practice. The first half of the training session extensively introduced the background and scoring instructions of all the self-reports to be administered to the adolescents. Each research assistant was tasked to familiarise with all items, content, and scoring instructions present within each self-report. This segment adequately prepares the research assistants for potential queries posted by the adolescent respondents when they attempt the self-reports. The second half of the session comprises training the administration of the semi-structured C-SSRS, including planning the safety management protocols if adolescents were to indicate high suicidal risk [42]. During this segment, research assistants took turns to roleplay as the adolescent or interviewer and each roleplay was assessed by author John C.M. Wong. The research assistant imitating a case subject may spontaneously indicate high suicidal risk, which tests the risk management skills of the other research assistant roleplaying as the interviewer. In such an event where high suicidal risk was detected during the interview, the research assistants would gently ask these adolescents if they would be comfortable receiving any formal support from healthcare professionals (i.e., hospital psychiatrists). Independent of their responses, the case would be highlighted immediately to the study team for the authors to inform the parent(s) about his/her suicidal risk. All participants would have consented to these procedures before commencing with the interview.

### Measures

#### Demographic variables

Self-reported information on demographic characteristics included age, sex, ethnicity, religious affiliation, level of education and housing information.

#### Suicide attempts

The semistructured interviewer-administered Columbia Suicide Severity Rating Scale (C-SSRS; [41]) consists of two subscales, each quantifying the severity of suicidal ideation and the intensity of suicidal behaviours respectively. Items from the “suicidal ideation” subscale were used to evaluate the severity and intensity of suicidal ideation. Similarly, items from the “suicidal behaviour” subscale were used to assess the presence of self-harm and lifetime/previous SAs. The single item on “actual attempts” obtained from the “suicidal behaviour” subscale was used as a screening tool to assess the presence of a recent SA (i.e. in the past 6 months, yes/no). Since this was the only item relevant in answering the study’s hypothesis, it was retained for further statistical analyses. The C-SSRS was widely known as the “gold standard” for suicidal risk assessment [43].

#### Temperament

Adolescents’ temperaments were assessed using the Revised Dimensions Of Temperament survey (DOTS-R; [44]), which is a 54-item self-report questionnaire that measures nine temperament dimensions as described in Table 1:

**Table 1:** Nine Dimensions of Temperament measured by DOTS-R

Subscale	Temperament Definition
Activity Level (General)	Assesses the extent of motor activity in the adolescent
Activity Level (Sleep)	Assesses the extent of motor activity during sleep
Rhythmicity (Sleep)	Assesses the regularity of the adolescent’s sleep-wake cycle
Rhythmicity (Eating)	Assesses the regularity of the adolescent’s eating habits (e.g. quantity of food)
Rhythmicity (Daily Habits)	Assesses the regularity of the adolescent’s daily routines (e.g., bowel movements, hunger).
Mood	High scores reflect a higher predisposition of positive affect (e.g., smiling and cheerful behaviours); Low scores reflect inherently anxious, depressive, and irritable behaviours.
Approach/Withdrawal	Assesses the adolescent’s comfort or hesitancy in approaching novel situations.
Flexibility/Rigidity	Assesses the adolescent’s adaptability to changes in his/her environment(s)
Task Orientation	Assesses the adolescent’s persistence or distractibility on a single task

Each of the nine dimensions uses a four-point likert scale ranging from 1 (*usually false*) to 4 (*usually true*). Higher scores reflected lower levels of activity, approach, adaptability, persistence, positive mood, and more regular eating and sleeping habits. The DOTS-R was known for its desirable psychometric properties, showing high convergent validity with other temperament measures [45], concurrent validity with personality traits [46], and excellent longitudinal stability across childhood and adolescence [47]. In our sample, all the nine subscales of the DOTS-R showed acceptable internal consistency. (α = 0.62 – 0.89).

#### Psychiatric disorders

The Youth Inventory-4 (YI-4 ; [48]) is a self-report rating scale that assesses symptoms of DSM-4 emotional and behavioural disorders in youths between 12 and 18 years old. The YI-4 contains 120 items that assess symptoms of 18 disorders, with each item rated on a four-point likert scale (0 = *never*, 3 = *very often*). The scale yields Symptom Count scores that are summed to derive criteria for diagnosis. The subscales for 8 diagnoses (yes/no), were used in the present study. MDD Comorbidity (yes/no) was determined when the adolescent met the symptom count criteria for another psychiatric disorder (i.e., Substance Use, Bulimia Nervosa, Generalised Anxiety Disorder, Schizophrenia, Dysthymia, Anorexia Nervosa) on top of MDD. The YI-4 previously demonstrated high reliability, showing consistency in its subscales with those of other diagnostic tools for youths (e.g., Youth Self-Report, [49, 50]). The internal consistency for all 8 symptom count scales in this study were excellent (α = 0.90 ~ 0.97).

#### Stressful life events

The Adolescent Stress Questionnaire (ASQ; [51]) consists of 58 items reflecting 10 common stress dimensions over the last 12 months. These are the stresses of (a) home life, (b) school performance, (c) school attendance, (d) romantic relationships, (e) peer pressure, (f) teacher interaction, (g) future uncertainty, (h) school/leisure conflict, (i) financial pressure, and k) emerging adult responsibility. Each participant rated the level of stress experienced on a 5-point likert scale (1 = not at all stressful to 5 = very stressful). The ASQ is widely regarded as a reliable and valid instrument in evaluating adolescent stressors from various sources [52–55]. In this study, the 10 subscales of ASQ showed excellent psychometric properties (α = 0.90–96).

#### Parental rejection

The Child Parental Acceptance-Rejection Questionnaire (PARQ; [56]) is a 60-item self-report questionnaire reflecting 4 subscales: warmth and affection (reverse scored), aggression and hostility, indifference and neglect, and undifferentiated rejection. Using a 4-point Likert scale, adolescents rated items from 1 = *almost never true* to 4 = *almost always true*, with higher scores indicating that s/he perceived the parent to be cold, aggressive, and/or neglecting. The PARQ has demonstrated high reliability across adolescent samples from 8 different countries, along with high discriminant validity of scores [57]. Coefficient alpha for both father and mother forms ranged from 0.86 to 0.92 for all subscales.

### Data analysis

All statistics were calculated using IBM SPSS v29.0 [58]. Student’s t-tests and chi-squared analyses were conducted to examine possible group differences in demographic variables, risk factors, and temperament traits. Binary logistic-regression models (i.e. Yes/No for Suicide Attempts) were used to determine the risk and protective temperament traits after adjustment of every risk factor (i.e., MDD/MDD comorbidity, stressful life events, parental rejection). To address the third hypothesis, we included the interaction term (i.e., Flexibility/Rigidity x Mood) in each of these models. To minimise multicollinearity, all variables were centred respectively onto their means and entered into the model simultaneously.

Independent variables for the logistic regression equation of the full model would consist of all demographic variables (age, sex, religion, education level, housing type), temperament traits (Activity Level General, Activity Level Sleep, Approach/Withdrawal, Flexibility/Rigidity, Mood, Rhythmicity (Sleep), Rhythmicity (Eating), Rhythmicity (Daily Habits), Task Orientation), the interaction term between “Flexibility/Rigidity” and “Mood” (Flexibility/Rigidity x Mood), perceived maternal and parental rejection (PARQ (Mother), PARQ (Father)), recent stressful life events during adolescence (ASQ), and MDD/MDD comorbidity.

## Results

### Preliminary analyses

Among the 60 cases, 43 adolescents (71.7%) indicated presence of suicidal ideation with 41 of them (68.3%) having suicidal thoughts for at least once a week. 23 case adolescents (38.3%) reported themselves as active suicidal ideators with a specific plan. 41 adolescents (68.3%) also previously attempted self-harm. 29 (48.3%) of the case adolescents were first-time attempters while the remaining 31 (51.7%) were multiple attempters. None of the 58 control adolescents showed any presence of suicidal ideation or any attempts of self-harm and suicide. In addition, there were no differences in age (t(187) = 0.925, p = 0.356) and sex (X2(1, N = 189) = 1.58, p = 0.210) between the 71 adolescents who did not participate and the 118 adolescents who remained in the study. However, both groups significantly differed on ethnicities (X2(3, N = 189) = 12.7, p = 0.005) where the 71 adolescents who did not participate were more likely to be Chinese as compared to other ethnicities.

### Risk factors of adolescent SAs

As detailed in Table 2, the average adolescent suicide attempter was significantly more likely to be psychiatrically diagnosed (t = -6.62, p < 0.001), with MDD alone (t = -6.64, p < 0.001) or comorbid with MDD (t = -7.03, p < 0.001), experience more stressful life events (t = -5.69, p < 0.001), more paternal (t = -2.79, p < 0.01) and maternal rejection (t = -4.06, p < 0.001). Compared to non-attempters, adolescent suicide attempters experienced significantly higher rates in nearly all disorders and recent stressors (all p < 0.05), except for bipolar disorder (t = -1.23, p = 0.001). Cases and Controls were not significantly different in any of the sociodemographic characteristics (all p > 0.05). In terms of temperament (Table 3), these cases scored significantly lower than controls on four “difficult” traits (Flexibility/Rigidity: t = 3.85, p < 0.001; Rhythmicity in Sleep: t = 2.98, p < 0.01, Rhythmicity in Eating: t = 1.92, p < 0.05; Mood: t = 4.88, p < 0.001; Approach/Withdrawal: t = 2.67, p < 0.001) and higher in Activity Level (General) (t = -1.63, p = 0.050).

**Table 2:** Differences in Clinical and Demographic Characteristics between adolescent suicide attempters and non-attempters

		Suicide Attempters *(N* = 60)		Non-Suicide Attempters (*N* = 58)		*p*
		Mean	*SD*	Mean	*SD*	
Age		16.40	2.00	16.0	1.68	0.121
ASQ (Total)		145.40	39.09	108.59	30.56	**< 0.001**
	Home Life	28.53	9.38	20.14	8.52	**< 0.001**
	School Performance	20.92	7.24	18.21	5.31	**< 0.05**
	School Attendance	7.97	3.73	5.74	2.65	**< 0.001**
	Romance	12.43	6.36	7.28	4.06	**< 0.001**
	Peer Pressure	19.97	7.18	13.7	5.44	**< 0.001**
	Teacher Interaction	14.57	6.57	11.02	3.67	**< 0.001**
	Future Uncertainty	10.72	3.10	8.57	2.95	**< 0.001**
	Social Conflict	13.52	5.37	11.16	4.58	**< 0.01**
	Finance Pressure	10.72	4.49	7.59	3.47	**< 0.001**
	Adult Responsibility	6.07	2.83	5.19	2.24	**< 0.05**
PARQ (Father)		138.52	35.71	118.96	38.99	**< 0.01**
PARQ (Mother)		120.73	37.37	96.6	25.85	**< 0.001**
		N	%	N	%	*p*
Sex						0.605
	Male	17	28.3	14	24.1	
	Female	43	71.7	44	75.9	
Ethnicity						0.942
	Chinese	29	48.3	31	53.4	
	Malay	13	21.7	11	19.0	
	Indian	15	25.0	14	24.1	
	Others	3	5.1	2	3.4	
Religion						0.278
	Buddhist/Taoist	6	10.0	11	19.0	
	Muslim	16	26.7	15	25.9	
	Christian	12	20.0	10	17.2	
	Hindu/Sikh	11	18.3	13	22.4	
	No Religion	15	16.7	9	15.5	
Education level						0.670
	High School	44	73.3	41	70.7	
	Diploma/Community College	16	26.7	17	29.3	
Housing Type						0.101
	1–2 room(s)	4	6.7	1	1.7	
	3 rooms	11	18.3	8	13.8	
	4–5 rooms	40	66.6	35	60.3	
	Private Housing	5	8.3	14	24.1	
Met Diagnostic Criteria for any Disorders		53	88.3	22	37.9	**< 0.001**
	Major Depressive Disorder	35	58.3	5	8.6	**< 0.001**
	Substance Use	10	16.7	2	3.4	**< 0.05**
	Bulimia Nervosa	19	31.7	1	1.7	**< 0.001**
	Generalised Anxiety Disorder	36	60.0	9	15.5	**< 0.001**
	Schizophrenia	15	25.0	1	1.7	**< 0.001**
	Dysthymia	44	73.3	9	15.5	**< 0.001**
	Bipolar Disorder	11	18.3	6	10.3	0.217
	Anorexia Nervosa	17	28.3	2	3.4	**< 0.001**
MDD Comorbidity		35	58.3	4	6.9	**< 0.001**

**Table 3:** Differences in Temperament Trait scores between adolescent suicide attempters and non-attempters

	Suicide Attempters *(N* = 60)		Non-Suicide Attempters (*N* = 58)		*p*
	Mean	*SD*	Mean	*SD*	
Activity Level (General)	21.45	4.26	20.09	4.81	**0.050**
Activity Level (Sleep)	10.65	2.97	10.45	3.37	0.365
Rhythmicity (Sleep)	11.28	3.37	13.40	4.30	**< 0.01**
Rhythmicity (Eating)	10.07	3.26	11.36	4.05	**< 0.05**
Rhythmicity (Daily Habits)	10.05	2.63	10.50	2.74	0.182
Mood	19.23	5.90	23.76	3.94	**< 0.001**
Flexibility/Rigidity (Adaptability)	12.13	2.73	14.19	3.08	**< 0.001**
Approach/Withdrawal	17.18	3.76	18.91	3.26	**< 0.01**
Task Orientation	18.55	4.54	19.6	3.78	0.167

### Odds ratios for adolescent suicide attempts

**Fig. 2:**
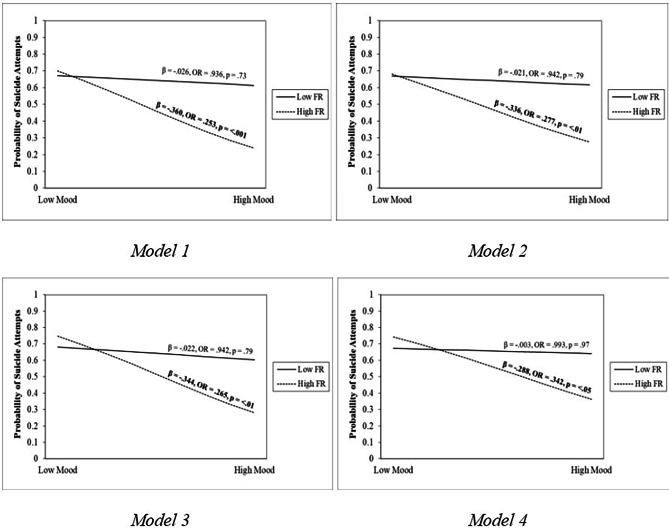
Probability of adolescent suicide attempts as a function of mood and flexibility/rigidity Functions are graphed for two levels of flexibility/rigidity: 1 standard deviation above the mean and 1 standard deviation below the mean. F/R = Flexibility/Rigidity Model 1 adjusted for all temperament traits and demographic variables Model 2 adjusted for all temperament traits, demographic variables, PARQ (Mother) and PARQ (Father) Model 3 adjusted for all temperament traits, demographic variables, PARQ (Mother) and PARQ (Father), and ASQ (Total) Model 4 adjusted for all temperament traits, demographic variables, PARQ (Mother) and PARQ (Father), ASQ (Total), and MDD Comorbidity

Table 4 displays the results of four binary logistic regression models with the “difficult” traits and the interaction term “Flexibility x Mood” as predictors of suicide attempts. The first model adjusted for demographic characteristics and the remaining traits, with the second, third, and fourth model additionally controlling for paternal and maternal rejection, stressful life events, and MDD comorbidity respectively. Since all but one adolescent in the control group met criteria for another psychiatric disorder (i.e., all except bipolar disorder) on top of MDD (Table 2), there was no point in controlling for MDD morbidity as a covariate. Therefore, the fourth model adjusted for MDD comorbidity together with the aforementioned factors. The fourth model, which controls all covariates, explained 59.1% (Nagelkerke R2 = 0.591) of the variance in SA and accurately classified 81.8% of the cases. Sensitivity and specificity of this model was 77.6% and 82.1% respectively. Across all four models, a unit decrease in “Mood” was significantly associated with a 12 − 18% (OR: 1.12–1.18, p = < 0.05) increase in the likelihood of suicide attempts. Even after controlling for all covariates, “Negative Mood” (OR: 1.18, p = < 0.05), MDD comorbidity (OR: 10.7, p = < 0.01), and stressful life events (OR: 1.02, p = < 0.05), remained as statistically significant predictors. Likewise, a unit increase in the interaction term “Flexibility x Mood” was significantly associated with a 5 − 6% decrease in the likelihood of suicide attempts (OR: 0.943 – 0.955, p = < 0.05) across all four models. This significant interaction was later probed at -1SD and + 1SD of the Flexibility/Rigidity (Adaptability) scores. Figure 2 visualised the gradual changes in the simple slopes after controlling for each additional risk factor. After adjustment of all temperament traits, demographic and risk factors, simple slope analyses revealed that “Positive Mood” significantly predicted lower probability of suicide attempts when “Adaptability” was high (b = − 0.288 – − 0.293, p < 0.05) but not low (b = − 0.003 – − 0.012, p > 0.87) (Model 4, Fig. 2).

**Table 4:** A logistic regression estimation results of adolescent’s temperaments with attempted suicide as a dependent variable, after controlling for individual differences

Temperament	Model 1		Model 2		Model 3		Model 4	
	β	OR (95% Cl)	β	OR (95% Cl)	β	OR (95% Cl)	β	OR (95% Cl)
Activity Level General	0.149	1.16* (1.03–1.32)	0.147	1.16* (1.02–1.32)	0.123	1.13 (0.987 − 1.30)	0.070	1.07 (0.928 − 1.24)
Approach/Withdrawal	− 0.065	0.937 (0.799 − 1.10)	− 0.100	0.905 (0.761 − 1.08)	− 0.095	0.909 (0.760 − 1.09)	− 0.051	0.951 (0.780 − 1.16)
Flexibility/Rigidity	− 0.142	0.867 (0.717 − 1.05)	− 0.113	0.893 (0.733 − 1.09)	− 0.006	0.994 (0.795 − 1.24)	− 0.055	0.946 (0.743 − 1.20)
Mood	− 0.201	0.818*** (0.727 − 0.921)	− 0.182	0.833** (0.740 − 0.939)	− 0.183	0.833** (0.736 − 0.942)	− 0.128	0.880* (0.773 − 0.998)
Rhythmicity Sleep	− 0.171	0.843* (0.710 − 0.973)	− 0.140	0.870 (0.727 − 1.04)	− 0.095	0.909 (0.757 − 1.09)	− 0.108	0.898 (0.735 − 1.10)
Rhythmicity Eating	− 0.002	0.998 (0.858 − 1.16)	− 0.041	1.04 (0.886 − 1.23)	− 0.060	1.06 (0.898 − 1.26)	− 0.075	1.08 (0.904 − 1.29)
Flexibility/Rigidity x Mood	− 0.059	0.943** (0.900 − 0.987)	− 0.054	0.948* (0.902 − 0.996)	− 0.053	0.949* (0.902 − 0.996)	− 0.046	0.955* (0.904 − 0.989)
PARQ (Father)	--	--	0.006	1.01 (0.991 − 1.02)	0.002	1.00 (0.986 − 1.02)	0.005	1.01 (0.987 − 1.02)
PARQ (Mother)	--	--	0.017	1.02* (0.980 − 0.996)	0.013	1.01 (0.994 − 1.03)	0.002	1.00 (0.981 − 1.02)
ASQ	--	--	--	--	0.019	1.02* (1.01–1.04)	0.019	1.02* (1.01–1.04)
MDD Comorbidity	--	--	--	--	--	--	2.37	10.7** (2.24–51.39)

*p < 0.05; **p < 0.01; ***p < 0.001

PARQ = Parental Acceptance and Rejection Questionnaire; ASQ = Adolescent Stress Questionnaire; MDD = Major Depressive Disorder; MDD Comorbidity = Presence of a second disorder on top of MDD

Models 1–4 adjusted for all demographic variables (i.e., age, sex, religion, education level, housing type) and other temperament traits (i.e., Activity Level Sleep, Rhythmicity (Daily Habits), Task Orientation). None of these variables were statistically significant (all p > 0.05)

Note: Multicollinearity diagnostics were assessed using the Values of Variance Inflation Factor (VIF), where a score below 2.5 indicates absence of multicollinearity. VIF values of all predictors were below 1.60, indicating our findings were unlikely due to spurious effects

## Discussion

This study sets out to investigate three main aims. Besides greater perceived parental rejection, higher frequencies of stressful life events, psychiatric disorders, and MDD comorbidity, we expected Asian adolescent suicide attempters to fare significantly worse than non-attempters on four “difficult temperament” traits (i.e., except Approach/Withdrawal). Our first hypothesis was only partially supported, such that suicide attempters were also significantly less approachable than non-suicide attempters. This finding contradicts previous observations that a high withdrawal temperament was protective against suicidality-related stressors among Asian adolescents. There are two possible reasons. With globalisation, education systems, school cultures, and parenting styles in traditionally collectivistic societies have shifted towards emphasising individualism [59]. The most recent studies of urban adolescents in China suggested robust negative relations between “withdrawal” and maladjustment [60–62], comparable to the findings of Western individualistic samples [63]. Due to the likeness between urban China and contemporary Singapore culture [64], similar inferences may apply to these equally independent Singaporean adolescents. Researchers are encouraged to replicate this observation with adolescent samples in other conservative parts of Asia (e.g., Korea). Otherwise, it is plausible that all five “difficult temperament” traits can be overrepresented even among Asian adolescent suicide attempters. While highly withdrawn Asian adolescents were generally better adjusted in school and at home, they were nonetheless disadvantaged in formal situations whereby they hesitate to participate in organised learning opportunities, teenage romance, and resolving serious peer-to-peer conflicts [65]. Such negative experiences could precipitate a cascade of self-esteem or self-competence issues [66]. Competitiveness in the contemporary Asian societies may then aggravate the effects of these internalising problems [67], strengthening the predictability of SAs. Together, this study found that adolescent suicide attempters significantly differed from non-attempters in scores favouring all five “difficult temperament” traits.

Our second hypothesis that the “Low/Negative Mood” trait remained a robust, significant predictor of adolescent SA was supported. Our study extended earlier findings by adjusting for additional risk factors that are important during this vulnerable stage of development (i.e., perceived parental rejection, proximal stressful life events, MDD Comorbidity). Adolescents who experienced more frequent, negative emotions were also inherently less tolerable of them, putting them at risk of using non-suicidal self-injury or SA as a maladaptive emotion regulation strategy [68]. This increased risk was also independent of their psychiatric disorders or stressful life events [20]. Our final hypothesis that the interaction between “Mood” and “Flexibility (Adaptability)” may be a possible pathway of buffering against SAs was also supported. This finding mirrors previous studies which demonstrated that a “hyperthymic” temperament uniquely reduced the likelihood of SAs even after controlling for sociodemographic, internalising and externalising disorders. While we may infer that the interplay between “Positive Mood” and “High Adaptability” underlies the protective role of a hyperthymic temperament, future researchers may further validate this suggestion using confirmatory factor analyses. Neuroimaging studies have somewhat supported this finding by revealing that “Positive Mood” and “Adaptability” temperaments share biological correlates with the personality trait resilience developed at late adolescence or young adulthood [69]. Irrespective of flexibility levels, adolescents low in mood were significantly more likely to attempt suicide (Fig. 2), further justifying our earlier results on “Negative Mood” as a direct risk factor. Notably, recent stressful life events (OR: 1.02) or MDD comorbidity (OR: 10.2) also retained their significance in predicting adolescent SAs.

### Implications

This study highlights the importance of early identification of temperament traits among Asian youths. Not only were the “difficult temperament” traits significantly overrepresented among suicide attempters in Asia, but also the temperament trait of “Negative Mood” should warrant greater attention as a robust predictor of adolescent SAs independent of other established high-risk factors. In a similar vein, our results on the interaction between “Positive Mood” and “High Flexibility” traits being a protective mechanism notably challenged previous speculations that a positive outlook alone was sufficient to reduce adolescent SAs. This finding is timely given that recent research was also identifying protective traits within the multifaceted hyperthymic temperament [36]. Yet, it may not be wise to proceed immediately with mandatory early screening of these “difficult” or “protective” traits, given that predictions of SAs have been dominated with false-positive and false-negative findings [70–72]. Practitioners are calling for a more measured and strategic approach to adolescent suicide prevention in order to minimise false-positive findings and maximise precision [73]. To do this, we suggest three possible directions that future researchers can undertake. One, future researchers should aim to converge on these findings even with employing different study designs, samples, and measurement types. For instance, using a 10-year prospective longitudinal study design with the administration of annual self-reports on Mexican-origin adolescents, Lawson et al. (2022) established consistent associations between the “Negative Emotionality” trait and active suicidal ideation and behaviours across their development from 13 to 22 years old [74]. Despite having administered a different temperament scale (i.e., Early Adolescent Temperament Questionnaire-Revised/EATQ-R; [75]), the “Negative Emotionality” subscale has shown good convergent validity with the “Negative Mood” trait assessed by the DOTS-R [76]. The confluence of both findings onto this “Negative Mood” trait gives confidence of its robustness in predicting suicidality-related behaviours among these adolescents regardless of culture. However, our last finding that the synergistic effects of high adaptability and positive mood temperaments were protective against adolescent SAs were not explicitly tested elsewhere. Without the support of other research methodologies, such as factor analyses to validate if these temperament traits truly underlie the protective “hyperthymic” profile, it may be ill-advised to proceed with screening for adolescents with high adaptability and emotionality and consider them to be “not at-risk” as this may result in false-negatives. Two, we suggest that collection of more neurobiological evidence (i.e., biological markers and correlates) linking adolescent temperaments and suicidal behaviours may be very helpful in objectively validating these connections. For example, few studies have already established strong relationships between different facets of a depressive temperament (e.g., fearfulness, pessimism; [77]), an impulsive temperament [78], and lower serotonergic activities, in which the latter was known to substantially increase the presence and lethality of SAs [79]. In a similar vein, we encourage biological researchers to test for common linkages between the neurobiology underlying a “hyperthymic” (or “High Flexibility” and “Positive Mood”) temperament and the protective neurobiological correlates of adolescent suicidal behaviours. Three, and in particular, adolescent suicide research still suffers from a serious lack of longitudinal data [74, 80]. This may be important considering that some developmental models of temperament and psychopathology (e.g., scar model; [81, 82]) postulated that experiencing suicidal ideation can lead to further changes in adolescent temperament. Longitudinal work that examines temperament development from an early age and all across adolescence may derive more insights on causality, pertaining to (i) whether temperament traits can lead to the onset of suicidal risk and if so, (ii) what are these traits and (iii) what were the contexts? We suggest that when there is sufficient research in these three directions, meaning that the developmental associations between these temperament traits and adolescent suicidal behaviours were consistent from a biopsychosocial perspective, there is greater conviction in temperament screening as an effective methodology in improving suicide prevention efforts (i.e., identifying true-positives while minimising false-positives). In this scenario, we may be more prepared to follow previous suggestions of implementing a collaborative network of school counsellors, paediatricians, and mental health care providers to partake in early screening of these “at-risk” temperaments [83]. Early identification of these traits could help to inform carefully tailored early interventions and prevent future SAs. For instance, to reduce direct influence of the “Negative Mood” trait on the likelihood of adolescent SAs, researchers and practitioners may consider the possibility of early CBT-skills training (e.g., cognitive restructuring) delivered in the form of youth-friendly psychoeducation for older children or early adolescents. This can help address cognitive vulnerabilities (i.e., hopelessness) that previously mediated the relationship between this temperament trait and adolescent SAs [84]. Such intervention has also worked well for “difficult” adolescents in coping with eating disorders [85]. In short, more research on intervention targets that mediate the effects of temperaments on SA may be important in tailoring effective early interventions [86]. Early detection of “at-risk” temperaments may also help in directing substantial support towards their parents as they raise these children/adolescents by means of increasing awareness of suicidal ideation and knowledge to mitigate suicidal risk before they intensify. The capacity to implement safety management procedures within the family can also greatly reduce the likelihood of a SA [87]. .

### Limitations and future directions

Several limitations of the current study should be acknowledged. First, our findings must be interpreted with caution as adolescents’ self-reports of their temperament, psychiatric symptoms, recent stressful life events, and perceived parental rejection may have been subjected to different kinds of biases. For example, adolescents may have been influenced by negative state emotions induced by their present psychiatric conditions or unstable cognitions associated with their admission in an emergency setting. Such negative mood or cognition may bias the responses of these adolescents in unpredictable ways [88]. Adolescents who meet the symptom criteria for specific disorders such as substance use or schizophrenia may also be suffering from memory or cognitive deficits which can further hamper the accuracy of self-reports [89, 90]. Social desirability biases could have also led to the under-reporting of mental disorders or symptoms by the adolescents. Future studies are encouraged to either minimise the use of self-reports or administer some of these scales (i.e., DOTS-R, PARQ) together with the parent/guardian and align their reports with the adolescent’s in order to reduce response biases by these affected adolescents. To minimise mood-dependent recall biases, we also encourage researchers to carry out the measures outside of the emergency setting. To our knowledge, the most robust developmental relations between temperament and suicidal behaviours were derived using prospective longitudinal designs, where researchers diligently monitored semi-annual/annual self-reports or interviews of temperament, suicidal behaviours, and demographical variables starting from early adolescence to young adulthood (e.g., [74]). Such prospective longitudinal designs with multiple waves of data collected over the period of adolescence may be optimal in depicting the development of suicidal risk in relation to temperament traits across time and should be considered for future studies. Second, self-reported temperaments could have been the reflections of recent states rather than traits. Nevertheless, temperament traits as measured by the DOTS-R have previously demonstrated excellent stability from early childhood to late adolescence in longitudinal studies [91], while trained research assistants who were present to clarify any queries from the respondent(s) may favour the assessment of traits more than states [92]. Third, there exists structural limitations within the self-reports used to measure adolescents’ temperaments (i.e., DOTS-R) and behavioural disorders in youths (YI-4R). Though the nine dimensions of temperament assessed by the DOTS-R were extensive [93], however, previous factor analytic research found that they were not entirely independent of each other. Rothbart and Mauro (1990) observed that the definition of “Positive/Negative Mood” overlapped with the definition of “Approach/Withdrawal” [94]. This may limit the implications that can be drawn from our findings. For instance, we cannot be certain if the predictive strength of the “Negative Mood” trait was due to some elements of “Withdrawal” correlated with negative emotionality. Yet, for clarity, the DOTS-R was carefully selected for this study due to two features that may be lesser known for other temperament measures [95]. One, the DOTS-R was tested to be highly invariant across cultures, demonstrating the universality of these nine dimensions of temperament. These nine dimensions showed structural consistency across adolescents of different cultures and ethnic groups [96, 97]. Recently, this instrument demonstrated high internal and external validity among 775 Chinese adolescents and was suggested to be optimal for assessing Asian adolescents’ temperament profiles [98]. Two, the DOTS-R demonstrated long-term predictive associations with some internalising and externalising disorders (i.e., psychiatric and substance use disorders; [12, 99]. Therefore, some researchers considered this scale to be one of the very few highly validated instruments for at-risk adolescents. These researchers suggested that the DOTS-R may help to detect developmental deviance at an early age as its broad dimensions of temperament sufficiently describe emotional or behavioural patterns that can lead to clinically relevant symptoms [100]. Nonetheless, to obtain less ambiguous conclusions [101], future studies may utilise other temperament assessments like the EATQ-R [75] which empirically categorises temperament into three distinctive superordinate factors — positive emotionality, negative emotionality, and self-regulation [102]. Next, the YI-4 was preferred to other reliable diagnostic tools such as the Mini International Neuropsychiatric Interview for Children and Adolescents (MINI-KID) due to primary concerns about the availability of the research assistants (interviewers) and the parent(s)/guardian(s) of the subjects. For the interviewers, administration of the YI-4 does not require any formal training except to instruct the adolescents to self-report their symptoms. Parental reports are not needed, while adolescents’ self-reported symptom counts were reviewed by a senior psychiatrist to give the diagnoses [48]. These procedures were helpful for our research assistants who were already assisting in various projects at that time, and may have prevented further attrition from the sample if the parent(s)/guardian(s) were also required to participate in the interviews. Consequently, we acknowledge that the YI-4 self-reports may have been influenced by recall biases or mood-dependent response biases associated with the adolescents’ psychiatric conditions or their unstable cognitions in an emergency setting [88]. These biases may be reduced using structured diagnostic interviews such as MINI-KID, which aligns the responses of both adolescent and parent/guardian typically present during a MINI-KID interview [103].Furthermore, these responses are separately evaluated by an interviewer who has received formal training on basic knowledge of all psychiatric disorders and independent rating skills [103, 104]. For these benefits, future researchers are encouraged to adopt these alternative measures to derive more objective findings. Fourth, independent of the psychiatric conditions already assessed using the YI-4, neurodevelopmental disorders such as attention-deficit hyperactivity disorder (ADHD) and autism spectrum disorder (ASD) which were heavily linked to suicidal behaviours were not evaluated and controlled for as covariates in the statistical models. This may have affected the validity of our findings and gives another reason why future researchers should consider structured clinical assessments such as the MINI-KID which comprehensively assesses all psychiatric and developmental disorders for children and adolescents. Fifth, our small sample size also did not allow for any meaningful comparisons between case adolescents with different history of suicidal behaviours (e.g., mild vs. active suicidal ideator, multiple attempters vs. single attempters) unlike other recent studies [105]. Future studies may wish to recruit larger samples and compare the differential associations between temperament and adolescent SA across suicide attempters with different backgrounds. Sixth, as the study team was mainly interested in exploring systematic differences between adolescent suicide attempters and healthy youths, there was no matching between cases and controls on the severity of medical conditions. It is possible that some of these cases suffered from more serious medical conditions than controls. Future researchers may match cases and controls based on similar medical conditions or control for these conditions as covariates to allow a cleaner comparison between both groups. Seventh, this study was conducted before the pandemic. Though temperament traits were broadly considered to be highly stable across time and that COVID-19 infections did not biologically affect early temperament development [106], this may nonetheless limit the applicability of our findings. For instance, stress and life disruptions associated with maternal COVID-19 infections may still result in maternal-rated changes in infant temperament at 6 months [107]. This may subsequently affect parental attitudes or upbringing towards these infants, which would then affect their development. Future researchers can attempt to replicate our findings during pandemic or even post-pandemic times. Eighth, there existed some difficulties in recruitment as all 59 adolescents who rejected the in-person interviews with the research assistants despite showing interest initially did not state any reasons for doing so. In view of the fact that suicidal behaviours are still heavily stigmatized, future studies may consider recruiting more mental health professionals to proactively assist with recruitment procedures. Ninth, demographics are limited to Singaporean youths, primarily at late adolescence, which limits the generalizability of our findings across all adolescents and those beyond Asia. Possibly, our individualistic, Singaporean sample may also not be representative of highly collectivistic Asian youths. Future studies may consider re-evaluating the relationship between temperaments and SAs with cross-cultural Asian youths. Lastly, the cross-sectional nature meant that we cannot conclude causality between the two. To validate these findings, researchers are highly encouraged to consider examining longitudinal associations between temperament and suicidal behaviours throughout adolescence (e.g., [74]).

## Conclusion

Overall, this study examined difficult temperament traits, especially “Negative Mood” trait, to be indirect and direct risk factors respectively among Asian adolescent suicide attempters. This study also examined the interactive relationship between “Positive Mood” and “Adaptability” temperament traits as a possible protective pathway against adolescent SAs. Our results advocate for early temperament screening as an important means to adolescent suicide prevention. Though, more longitudinal and neurobiological research converging on these temperament findings may be necessary before ascertaining temperament screening as an effective methodology to improve suicide prevention efforts for adolescents.

## Data Availability

The datasets generated and/or analysed during the current study are available from the corresponding author(s) on reasonable requests.
